# Regulation of Intrinsic Functions of PD-L1 by Post-Translational Modification in Tumors

**DOI:** 10.3389/fonc.2022.825284

**Published:** 2022-03-23

**Authors:** Naoe Taira Nihira, Yoshio Miki

**Affiliations:** ^1^ Department of Molecular Genetics, Medical Research Institute, Tokyo Medical and Dental University, Tokyo, Japan; ^2^ Department of Translational Oncology, St. Marianna University Graduate School of Medicine, Kawasaki, Japan

**Keywords:** immune checkpoint, post-translational modification, transcription factor, immunotherapy, PD-L1

## Abstract

Tumor cells are eliminated by the immune system, including T lymphocytes and natural killer cells; however, many types of tumor cells acquire the immune tolerance by inhibiting T-cell activation and functions *via* immune checkpoint molecules. Immunotherapy targeting immune checkpoint molecules such as Programmed death receptor 1 (PD-1)/Programmed death ligand 1 (PD-L1) and cytotoxic T lymphocyte associated protein 4 (CTLA-4) have shown successful outcomes for multiple cancer treatments, however some patients show the lack of durable responses. Thus, discovering the chemical compounds or drugs manipulating the expression or function of immune checkpoint molecules are anticipated to overcome the drug resistance of immune checkpoint inhibitors. Function of inhibitory immune checkpoint molecules is often dysregulated by the transcriptional and post-translational levels in tumors. Here, this review focuses on the post-translational modification of intrinsic PD-L1 functions and regulators for PD-L1 transcription.

## Introduction

**Figure 1:**
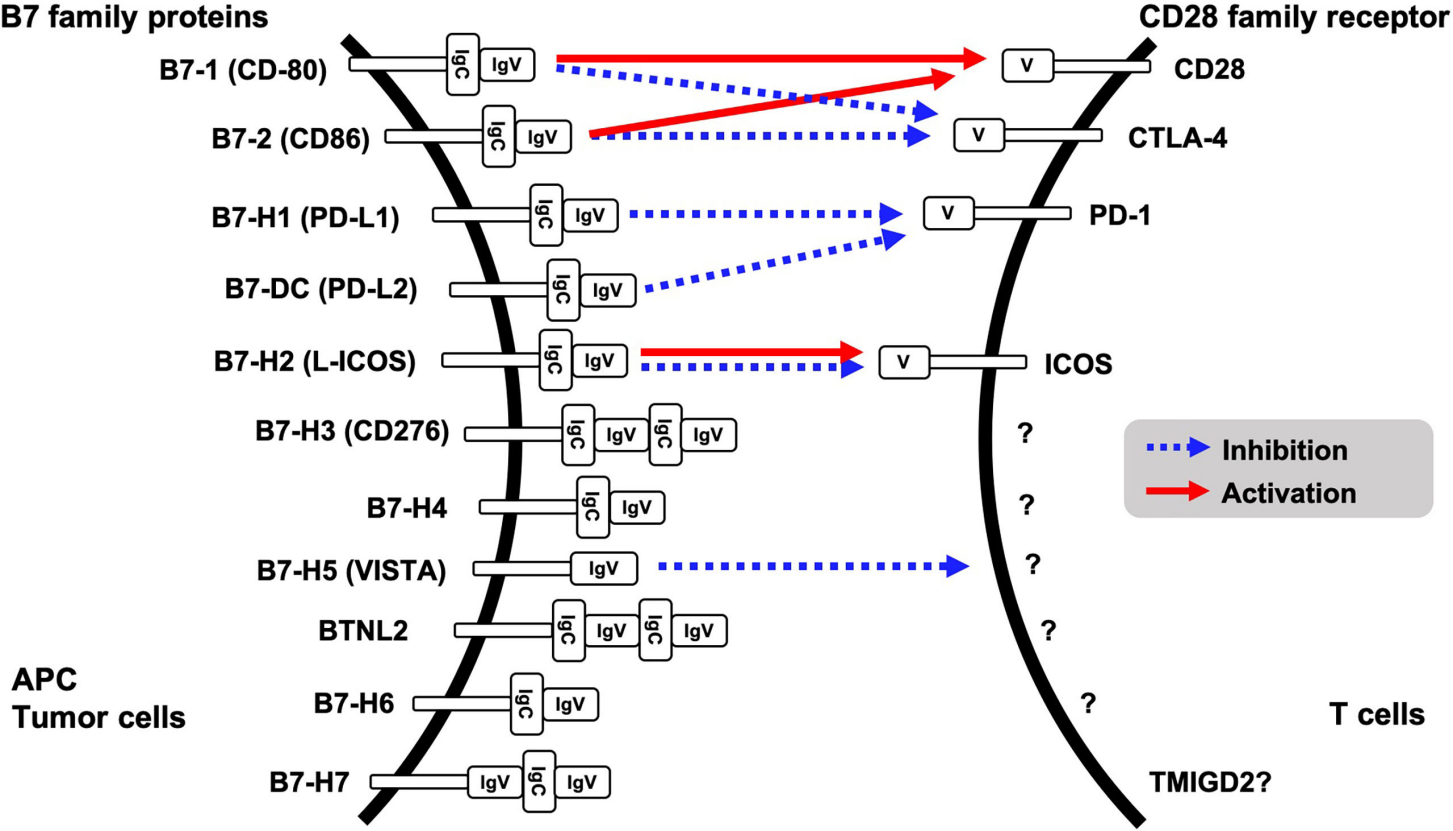
B7 family proteins and its binding receptors.

Evasion of innate immune response is one of the features of tumorigenesis and acquisition of resistance to cancer treatment. Binding of CD28 family receptors such as CD28, CTLA-4, PD-1 and ICOS on active T cells with B7 family ligands on antigen presenting cells and tumor cells triggers negative signal transduction for T cells, resulting in attenuated cell activation and growth. Currently, eleven B7 family ligands have been identified: B7-1, B7-2, B7-H1, B7-DC, B7-H2, B7-H3, B7-H4, B7-H5, BTNL2, B7-H6, and B7-H7. PD-L1 (B7-H1) and PD-L2 (B7-DC) are ligands for PD-1 on T cells (**Figure 1**). Intercellular interaction of PD-L1 on cancer cells with PD-1 on immune cells triggers tyrosine phosphorylation of the PD-1 cytoplasmic domain, resulting in reduced phosphorylation of TCR signaling molecules and inhibition of T cell activation and cytokine production (1). PD-L1 expression is upregulated in many different solid tumors, namely, colorectal cancer, hepatocellular cancer, lung cancer, gastric cancer, pancreatic cancer, and ovarian cancer. High expression of PD-L1 was associated with worse overall survival in cervical cancer, esophageal cancer, gastric cancer, non-small cell lung cancer, and ovarian cancer (2). Other CD28 family receptors, CD80 (B7-1) and CD86 (B7-2) have an affinity to CD28 or CTLA-4, and L-ICOS (B7-H2) functions as a ligand for ICOS on T cells. However, a binding receptor for B7-H3, B7-H4, BTNL2, B7-H6 and B7-H7 is unidentified. Among the B7 family protein, VISTA (B7-H5) has a unique character, because it might function both as co-inhibitory ligand and receptor. Although the other B7 family ligands harbor both IgV- and IgC-like domains in their extracellular regions, VISTA however contains a single IgV-like domain similar to the CD28 family receptors. Receptor on T cells for VISTA is currently unidentified; however overexpression of VISTA on bone marrow-derived dendritic cells triggered the inhibition of T cell proliferation and cytokine production, supporting VISTA function as the co-inhibitory ligand (3).

Blockade of the binding by immune checkpoint inhibitors such as antibodies against immune checkpoint molecules can inhibit the immune tolerance. Indeed, clinical studies of the therapeutic antibodies against PD-1 or PD-L1 showed successful outcomes in renal cell carcinoma, non-small cell lung cancer and ovarian cancer. Therefore, a number of antibodies against PD-1 and PD-L1 are developed by multiple pharmaceutical companies and are approved in clinical trials for melanoma, non-small cell lung cancer, head and neck squamous cell carcinomas, classical Hodgkin lymphoma and Merkel cell carcinoma by the U.S. Food and Drug Administration (FDA) (4). Recently, combination therapy with immune checkpoint inhibitors and the molecular target drugs might improve the efficacy. Molecular mechanism regulating expression or function of immune checkpoint molecules is a preferable target for the drug discovery. Here, we focus on the post-translational modifications regulating PD-L1 expression and its cellular function. This review provides insights into the establishment of new therapeutic strategies for immune therapy targeting PD-L1.

## Regulation of PD-L1 Expression

PD-L1 expression is tightly regulated in multiple layers of expression control: transcriptional upregulation by Myc, NF-κB and STAT1/3, inhibition of translation by miR34a which is induced by p53, ubiquitination by tumor suppressive ubiquitin E3 ligase SPOP, and de-ubiquitination by CSN5 (5, 6). At the transcription level, genotoxic stress and cytokines strongly increase PD-L1 expression.

### Genotoxic Stress

DNA damage stimuli such as irradiation, camptothecin, aphidicolin, and etoposide increase PD-L1 expression at the transcription level (7). Interestingly, adriamycin treatment decreases PD-L1 expression on plasma membrane and upregulates its nuclear expression (8). The activation of DNA damage response is essential to maintain genomic stability. DNA lesions are recognized by damage sensor molecules, Ataxia Telangiectasia Mutated (ATM) and Ataxia Telangiectasia and Rad3 Related Protein (ATR), and the damage signal is transduced into their downstream substrates, checkpoint kinase 1/2 (CHK1/2) and p53. DNA double-strand breaks (DSBs) and single-strand breaks (SSBs) are removed through two major repair pathways, Homologous recombination (HR) and non-homologous end-joining (NHEJ), respectively. Poly (ADP-ribose) polymerases 1 (PARP-1) binds to strand break-containing repair intermediates and catalyzes polymerization of ADP-ribose moieties from nicotinamide adenine dinucleotide (NAD^+^), resulting in the recruitment of DNA repair molecules, XRCC1, DNA ligase III, and DNA polymerase β (pol β), to DNA damage sites. Inhibition of PARP activity fails to recruit the repair molecules that trigger cell death due to DNA repair failure. Mechanistically, DNA double-strand break repair pathway activates STAT1 and STAT3 signaling and activated IRF1 transactivates PD-L1 gene (7). Indeed, phosphorylation of STAT1 at Tyr701 and Ser727 and STAT3 at Tyr705 and Ser727 is triggered by DNA damage such as irradiation and treatment of genotoxic agents, showing that DNA damage-induced PD-L1 transcription by JAK1/2-STAT1/2/3-IRF1 pathway (7).

Interestingly, PARP inhibitor-triggered GSK3β inactivation upregulates PD-L1 transcription in triple negative breast cancer cells (9). An increased expression of PD-L1 by PARP inhibitor enhances the immunosuppression efficacy against anti-PD-L1 antibody. The molecular mechanism underlying GSK3β that inhibits PD-L1 transcription is still unclear. GSK3β often functions as a priming kinase to facilitate poly-ubiquitination of its substrates such as c-Myc and c-Jun oncoproteins and proteasomal degradation (10). c-Jun forms the AP-1 complex with JunB and has a hyperactivity in Hodgkin lymphoma cells (11). Epstein–Barr virus (EBV) triggers recruitment of c-Jun and JunB to AP-1 response element in the enhancer region of PD-L1 gene, resulting in the transcriptional induction of PD-L1 (12). Transcription factor c-Myc also directly binds to promoter of PD-L1 gene (13). These findings suggested that the inactivation of GSK3β escapes c-Jun and c-Myc from proteasomal degradation, and the stabilization of c-Jun and c-Myc may induce PD-L1 expression. Not only in PARP-1 inhibition but CHK1 inhibitor also increases PD-L1 expression. Cytoplasmic DNA triggered by CHK1 inhibitor activates the STING pathway, an innate immune pathway and promotes anti-tumor immunity (14). Within the pathway, Cyclic guanosine monophosphate (GMP)-adenosine monophosphate (AMP) synthase (cGAS) recognizes cytoplasmic DNA and triggers the production of cyclic GMP-AMP (cGAMP), which binds and activates the adaptor protein STING as the second messenger. Binding of cGAMP with STING induces phosphorylation of IRF3 *via* TBK1, and IRF3, results in transcriptional activation of inflammatory genes in the nucleus. IRF forms a complex with p65 NF-κB on promoter region of PD-L1 gene to induce the transcription (15).

### IFN Stimuli

Both type I and type II interferon strongly induce PD-L1 transcription (16). Interferon gamma (IFNγ) is a member of the type II interferon family and a strong inducer for PD-L1 transcription. In human lung cancer cells, interferon regulatory factor 1 (IRF1) binds to the putative binding sequence in PD-L1 promoter region and induces the transcription depending on JAL/STAT pathway response to IFNγ (17). In melanomas (18), renal cell carcinomas (RCC) and squamous cell carcinomas of the head and neck (SCCHN), phosphorylated STAT1, and not STAT3, is involved in IFNγ-triggered PD-L1 transcription (16). In addition to IFNγ, IFNα has been also reported to induce PD-L1 expression (19). IFNα stimulation triggers phosphorylation of p38 and STAT3. Inhibitors of p38 and STAT3 reduce PD-L1 expression, therefore IFNα-mediated PD-L1 transcription is triggered through the STAT3 and p38 signaling pathways (20). Not only IFNα and IFNγ, but other cytokines such as IL-1*α*, IL-10, IL-27, and IL-32*γ* also trigger PD-L1 transcription in tumor cells (16, 21). In the presence of IL-27, STAT1, and not STAT3, promotes the PD-L1 transcription, and p65 is essential for IL-1*α*-mediated PD-L1 transcription (16).

### Other Stimuli

Autophagy is the non-inflammatory programmed cell death, and the essential processes to maintain the cellular homeostasis. The process of autophagy is divided into 4 steps: the autophagosome formation, the fusion with lysosome to form autolysosome and the degradation of cellular proteins and organelles. Intriguingly, autophagy induction attenuates PD-L1 expression (22). In human gastric cancer cells, the knockdown of ATG5 or ATG7 upregulated PD-L1 expression. Pharmacological inhibition of autophagy by chloroquine and bafilomycin A1 also significantly increased PD-L1 expression. Intriguingly, the increases were linked to NF-κB signaling activation (22). Consistently, Atg5 deletion mice showed the increased expression of PD-L1 *via* TBK1 activation (18).

## Intrinsic Functions of PD-L1

### Transcriptional Regulation

PD-L1 predominantly expresses on the plasma membrane as a ligand for PD-L1, where on the contrary, a small portion of PD-L1 located in the nucleus was reported in 2010 (8). However, the functional role of nucleic PD-L1 had not been clarified for long periods. As of the latest our study focused on the nuclear function of PD-L1 which demonstrated that PD-L1 interacts with DNA and transcription factors such as p65/RelA and IRF proteins to orchestrate gene expression (18). RNA-seq analysis showed that transcription of immune response-related genes, such as type I interferon signaling pathway, INF-γ mediated signaling pathway, NF-κB signaling and antigen processing and presentation *via* MHC I pathways, are downregulated in PD-L1 knockout (KO) cells (23). It suggested that PD-L1 might contribute to the observed sensitivity for anti-PD-1/PD-L1 treatment in patients with high PD-L1 expression though the transactivation of inflammation pathways and the increased neoantigen presentation. In addition to the inflammation-stimulating function of PD-L1 on the immune response, RNA-seq also clarified that PD-L1 role in acquisition of immune tolerance through the transcriptional induction of immune checkpoint factors, PD-L2, VISTA, and B7-H3 (23). Since these checkpoint molecules also inhibit T-cell activity, the transcriptional induction of these genes by nuclear PD-L1 might be an alternative mechanism for escaping from immune surveillance. In addition to the aspect of tumor immunity, PD-L1 is also involved in the transcriptional regulation of autophagy and mTOR-related genes (24).

The nuclear translocation is mainly regulated by post-translational modification within the cytoplasmic domain of PD-L1. The p300 protein acetyltransferase and Histone deacetylase 2 (HDAC2) are the regulators for the nuclear translocation. Deacetylated PD-L1 bind with Adaptin β2, Clathrin heavy chain and HIP1R through endocytosis. Subsequently, interaction of Vimentin and Importin α1 with PD-L1 enabled it to move through the cytoskeleton and translocate into the nucleus (23). Therefore, HDAC2 inhibitor blocks the nuclear translocation and function in transcriptional control. Notably, a combination of HDAC2 inhibitor, Santacruzamate A, with therapeutic antibody against PD-1 improved overall survival compared with either treatment alone (23). These findings suggested that combining HDAC2 inhibition with PD-1/PD-L1 blockade is a promising immunotherapeutic approach for cancer treatment.

### Interfering the Cell Signaling Pathways

#### IFN Signaling

The IFN plays critical anti-tumor effects such as apoptosis induction, inhibition of cell growth, and induction of cell senescence toward cancer cells. STAT3 is a transcription factor and functions as an anti-apoptotic molecule in cancer cells. IFN-triggered STAT3 phosphorylation at Tyr 705, which induces dimerization, nuclear translocation and DNA binding, was significantly increased in PD-L1 KO mice (25). In addition, Caspase-7 expression after being treated with IFN was also upregulated in the KO mice, suggesting the inhibitory role of PD-L1 on STAT3 and Caspase-7 activation triggered by IFN. The RMLDVEKC motif in cytoplasmic domain within PD-L1 is critical for the inhibition of IFN-mediated cytotoxicity, and somatic mutations, D276H and K280N, in the motif are reported in human carcinomas (25). Since the expression level of PD-L1 on plasma membrane is induced by IFN as written in *IFN Stimuli*, the downregulation of STAT3/Caspase-7 might be a negative feedback mechanism for IFN stimuli.

#### mTOR Signaling

A serine/threonine kinase mTOR forms two distinct protein complexes, mTORC1 and mTORC2, as a catalytic subunit and plays fundamental roles in protein synthesis, lipid and nucleotide synthesis, metabolism and autophagy. Reduction of PD-L1 expression reduced mTOR activity (24, 26); furthermore, PD-L1 blockade by antibody attenuated mTOR activity and glycolytic metabolism in tumor cells (27). Mechanistically, loss of PD-L1 decreased the phosphorylation of p70S6K Thr389 by mTORC1, and Akt Ser473 by mTORC2 in human ovarian cancer cells (24). Moreover, since mTORC1 inhibits autophagosome elongation and maturation, PD-L1 inhibits the autophagy induction in human and murine cancer cells (see the next section) (24, 26). Notably, PD-L1 attenuation enhanced the anti-proliferative effects of mTORC1 inhibitor rapamycin. Therefore, the combination of the mTOR inhibitor and therapeutic antibody against PD-L1 might improve the anti-tumor efficacy.

### Cell Death

Cell death is essential machinery for morphogenesis and maintenance of homeostasis, thus, dysregulation of the machinery might be a cause of human diseases, namely, cancers and autoimmunity. Types of cell death are divided into two major groups: non-programed cell death and programmed cell death. The programmed cell death can be categized into non-inflammatory manner and pro-inflammatory manner.

#### Autophagy

As described above, autophagy induction decreases PD-L1 expression. In contrary, PD-L1 depletion showed the increased autophagosome formation through modifying the transcription of autophagy induction-related genes. Notably, tumor cells with lower levels of PD-L1 expression showed resistance to autophagy inhibitor, chloroquine, comparing cells with highly expressed PD-L1 in both human and murine cancer cell lines (24). Therefore, expression status of PD-L1 in tumors might be a biomarker to autophagy inhibitor response. PD-L1 also inhibits the autophagic cytoskeleton collapse through interaction with Akt, triggering glioblastoma invasion (26). In glioblastoma tissue, energy-deprivation stress due to ischemia is closely linked with glioblastoma cell growth and invasion. PD-L1 expression did not affect the phosphorylation status of Akt at Ser473 and mTOR at Ser2448, which are biomarkers for activation of PI3/Akt pathway, under normal culture condition, although PD-L1 overexpression increased the phosphorylation upon serum starvation condition. Moreover, knockout of PD-L1 increased autophagic puncta formation pf Atg8/LC3 under serum deprivation conditions. Mechanistically, PD-L1 interacts with Akt and anchors it on the plasma membrane to activate PI3/Akt signaling pathway. In addition to the effect on cell distribution of Akt, PD-L1 affected the expression of cell migration, actin-structure functions, PI3K–Akt pathway and the regulation of actin cytoskeleton-related genes, resulting in inhibition of autophagy (26). As described in *Other Stimuli*, induction of autophagy by chloroquine and bafilomycin A1 attenuates PD-L1 expression (22). Therefore, these findings suggested that the reciprocal negative regulation between autophagy induction and PD-L1 expression.

#### Pyroptosis

Pyroptosis is pro-inflammatory programmed cell death, and triggered by the inflammatory subfamily of caspases and pyroptosis executioner Gasdermin D. Pyroptosis was originally identified in inflammatory cells such as macrophages and trigged by bacterial or pathogen infections. PD-L1 function on pyroptosis induction is poorly understood up until now. Intriguingly, hypoxia stress triggers the interaction of p-STAT3 at Y705 with PD-L1 in the nucleus and upregulates *Gasdermin C* expression, resulting in induction of caspase-8-mediated pyroptosis. Notably, nuclear PD-L1-induced pyroptotic cell death was diminished by treatment with a selective STAT3 inhibitor, HO-3867 (28). Thus, nuclear PD-L1 has a role as switch to promote pyroptosis under hypoxia stress condition.

### Regulation of mRNA Stability

As described in *Genotoxic Stress*, DNA damage is a strong trigger for PD-L1 transcription; however the intracellular functions of PD-L1 had been poorly understood. Intriguingly, Tu et al. showed that intracellular PD-L1 binds with mRNA of DNA damage-related genes such as BRCA1 and NBS1 under genotoxic stress and stabilize it due to the competition with the RNA exosome (29). Interestingly, PD-L1 antibody H1A destabilizes PD-L1 and the mRNAs, resulting in increased sensitivity to DNA damage.

## Conclusion and Perspectives

**Figure 2:**
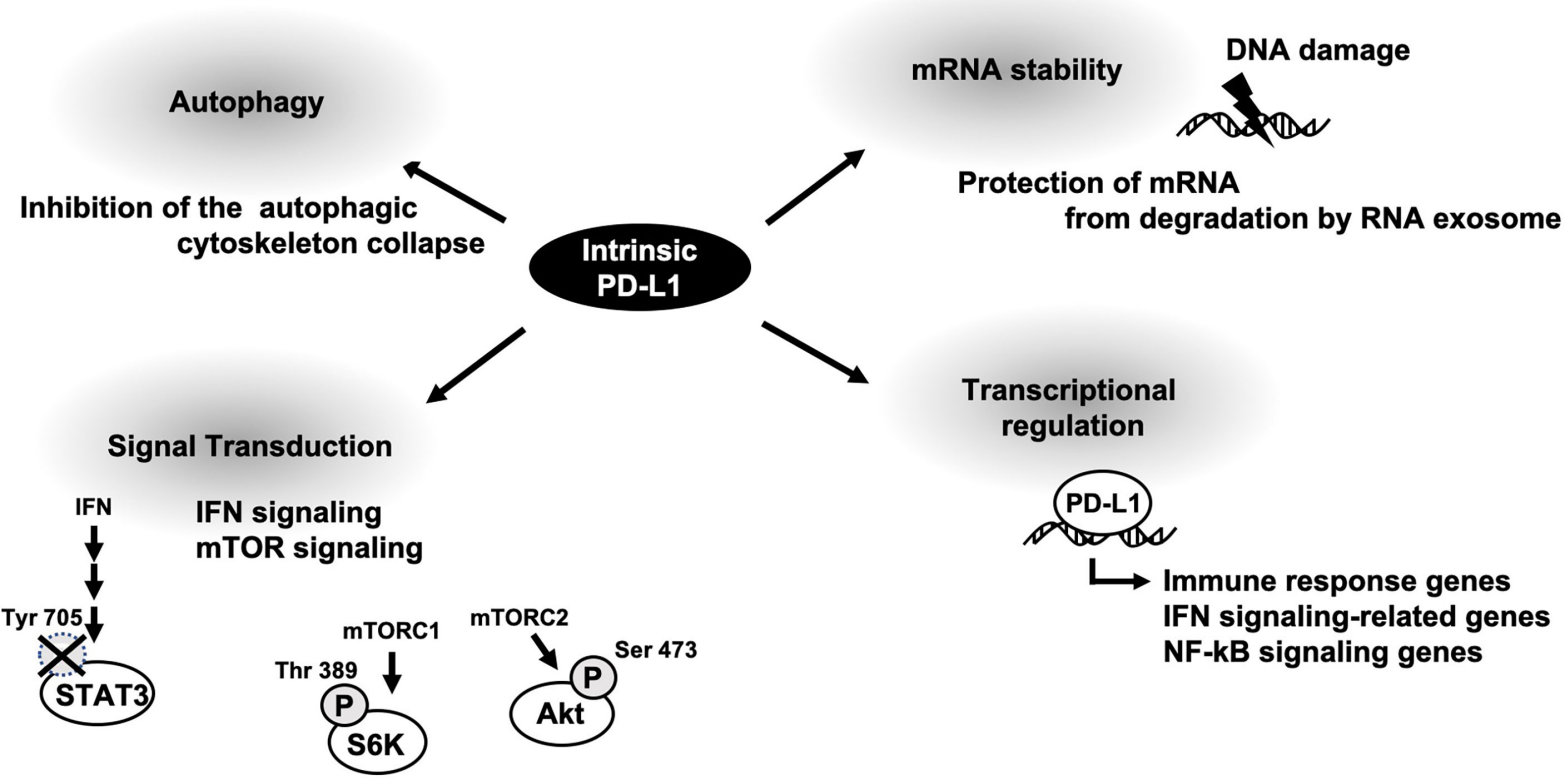
A schematic diagram of cellular PD-L1 functions.

Oncogenic functions of membrane PD-L1 has been well-understood by a variety of basic research and clinical studies. Intrinsic PD-L1 function had been poorly characterized; however some papers are now u ering it. As described above, cellular PD-L1 has more fundamental roles, not only tumor-promoting function (**Figure 2**). It might be on account of PD-L1 not being limited to express on tumor cells and some fundamental functions of intrinsic PD-L1 are common in both tumor cells and antigen presenting cells. Thus, selective inhibitors against tumor-promoting function of intracellular PD-L1 might be applicable to immune therapy. To this end, further investigation of intrinsic functions of PD-L1 is awaited.

## Author Contributions

NTN and YM contributed to the conception of this review and wrote the manuscript. All authors listed have made a substantial, direct, and intellectual contribution to the work and approved it for publication.

## Funding

NTN reports receiving grants from the Japan Society for Promotion of Science (JSPS) KAKENHI Grant (20J40010 to NTN); the Basic Science Research Projects from The Sumitomo Foundation; the Sagawa Foundation for Promotion of Cancer Research and the Mochida Memorial Foundation for Medical and Pharmaceutical Research. NTN is supported by JSPS Research Fellowships for Young Scientists.

## Conflict of Interest

The authors declare that the research was conducted in the absence of any commercial or financial relationships that could be construed as a potential conflict of interest.

## Publisher’s Note

All claims expressed in this article are solely those of the authors and do not necessarily represent those of their affiliated organizations, or those of the publisher, the editors and the reviewers. Any product that may be evaluated in this article, or claim that may be made by its manufacturer, is not guaranteed or endorsed by the publisher.
